# Interaction of CYP2C19 with the effect of clopidogrel in secondary prevention of major ischemic events: Systematic review and meta-analysis

**DOI:** 10.1177/17474930251372371

**Published:** 2025-08-22

**Authors:** Femke CC Kremers, Jochem van den Biggelaar, Hester F Lingsma, Ron HN van Schaik, Bob Roozenbeek, Diederik WJ Dippel

**Affiliations:** 1 Department of Neurology, Erasmus MC University Medical Center, Rotterdam, The Netherlands; 2 Department of Public Health, Erasmus MC University Medical Center, Rotterdam, The Netherlands; 3 Department of Clinical Chemistry, Erasmus MC University Medical Center, Dr. Molewaterplein 40, 3015 GD, Rotterdam, The Netherlands

**Keywords:** Antiplatelets, clopidogrel, CYP2C19, genotype-guided treatment, Ischemic stroke, major vascular event, meta-analysis, prasugrel, stroke, systematic review, TIA, ticagrelor

## Abstract

**Background and Aims:**

Clopidogrel may be a less effective antiplatelet agent for secondary prevention after cardiovascular events in carriers of the CYP2C19 Loss of Function (LoF) allele. Randomized controlled trials (RCTs) of clopidogrel in patients with known CYP2C19 carrier status have provided inconsistent results. This meta-analysis aims to pool evidence on the effect of different antiplatelet strategies on outcomes according to CYP2C19 LoF status.

**Methods:**

We conducted a systematic review and meta-analysis of RCTs to evaluate the interaction of CYP2C19 LoF allele on clopidogrel versus placebo or other antiplatelet agents in patients with cardiovascular disease or transient ischemic attack (TIA) or ischemic stroke. Primary outcomes were major adverse cardiovascular events (MACEs) including ischemic stroke, with major bleeding events assessed as a safety outcome. Random effects analysis estimated pooled odds ratios for LoF carriers and non-carriers.

**Results:**

Fifteen RCTs with 35,189 participants in total were included. When all interaction effects are pooled, the occurrence of MACE was 1.29 times higher in LoF variant carriers compared to non-carriers for clopidogrel treatment (p-interaction = 0.01). Risk of MACE was 1.20 times higher in LoF carriers compared to non-carriers when clopidogrel was compared to placebo (p-interaction = 0.13). In TIA or minor stroke patients, the interaction effect was 1.63 times larger (p-interaction = 0.02). Clopidogrel was less effective than prasugrel for MACE occurrence (1.57 times higher, p-interaction = 0.02) and ticagrelor (1.21 times higher, p-interaction = 0.19) in CYP2C19 LoF variant carriers. Bleeding outcomes were similar across groups.

**Conclusion:**

Clopidogrel is less effective in patients with CYP2C19 LoF genotype and cardiovascular disease, minor stroke, or TIA. The size and direction of the interaction warrant further research into the role of LoF genotypes and the cost-effectiveness of genetic testing. Prasugrel may be a more effective alternative for CYP2C19 LoF carriers.

**Registration—URL:**

https://www.crd.york.ac.uk/prospero/; Unique identifier: CRD42021242993.

## Graphical abstract

**Graphical abstract:**
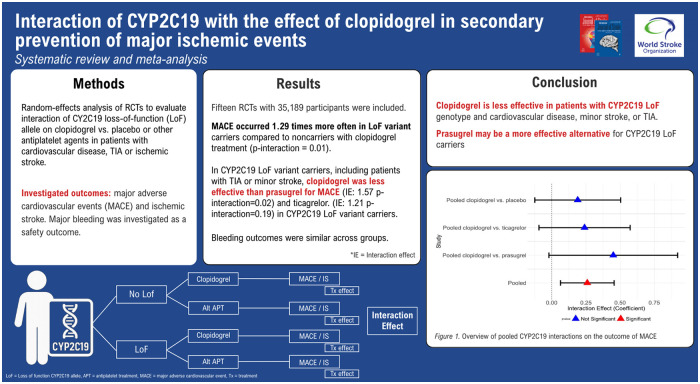

## Introduction

Patients with a history of ischemic stroke or cardiovascular disease can have recurrent ischemic events despite treatment with antiplatelet agents.^1,2^ This may partly be due to the incomplete effectiveness of these therapies. Some patients are unable to convert the clopidogrel prodrug to its active metabolite. This conversion is regulated by the hepatic cytochrome p450 enzymes, of which hepatic cytochrome p450 2C19 (CYP2C19) is an important contributor.^3^

Multiple variant alleles of the *CYP2C19* gene are associated with different levels of activity of the CYP2C19 enzyme. The *17 allele variant (-806 T > C; rs12248560) is associated with an increased CYP2C19 activity (Gain-of-function), whereas the *2 (681G > A; rs4244285) and *3 (636G > A; rs4986893) variants are loss-of-function (LoF) alleles and produce a CYP2C19 enzyme with decreased antiplatelet activity. Individuals carrying *2 or *3 alleles are called “Loss of function (LoF) carriers” and have a reduced clopidogrel metabolism.^4^ These LoF genetic variants occur in approximately 30% of the Western population and in up to 60% of the Asian population.^3,5,6^

There is little direct evidence of the interaction of CYP2C19 variants with antiplatelet agents on recurrent vascular events. In the randomized controlled trials (RCTs) that investigated whether clopidogrel combined with aspirin is superior to aspirin monotherapy for the prevention of new ischemic events, genetic substudies investigated the influence of *CYP2C19* LoF variants on the effect of clopidogrel treatment.^7^ Other alternative antiplatelet agents such as prasugrel and ticagrelor and their interaction with *CYP2C19* LoF variants have also been investigated.^8^ The results of these genetic substudies vary, and several studies were not sufficiently powered to detect a difference in effect between subgroups of patients with *CYP2C19* LoF variants.^9^

To further investigate this topic, we performed a systematic review and meta-analysis to estimate the effect of the *CYP2C19* LoF variants on the effect of different antiplatelet strategies in patients with cardiovascular ischemic disease, a recent transient ischemic attack (TIA), or minor ischemic stroke.

## Methods

### Search strategy

We performed a comprehensive literature search in the online databases Embase, Medline ALL, Web of Science Core Collection, Cochrane Central Register of Controlled Trials (CENTRAL) and Google Scholar on February 19, 2025. The search included RCTs that investigated the following; patients with cerebrovascular or cardiovascular disease treated with clopidogrel versus placebo or other P2Y12-inhibitors as secondary prevention and were followed for the occurrence of IS, major bleeding or a composite outcome for major cardiovascular events (MACE) of IS, myocardial infarction (MI), non-central nervous system systemic embolism, or vascular death (Supplementary Material I). In the present meta-analysis, we also included trials in patients with cardiovascular disease, such as acute coronary syndrome (ACS), atrial fibrillation (AF), and patients with multiple cardiovascular risk factors, who are at increased risk of recurrent ischemic events. In these studies, CYP2C19 status was assessed by genotyping the participants’ DNA.

This systematic review was registered with PROSPERO, the International Prospective Registry of Systematic Reviews (CRD42021242993).^10^ We performed this systematic review in accordance with the Preferred Reporting Items for Systematic Review and Meta-Analysis (PRISMA) guidelines,^11^ and we filled out the checklist (Supplementary Table I).

#### Outcomes

The investigated outcomes were (1) occurrence of fatal or non-fatal ischemic stroke or (2) MACE, IS, MI, non-central nervous system embolism, or vascular death. If a study did not distinguish between ischemic stroke and intracerebral hemorrhage, the study was not included in the analysis. Studies that did not describe outcomes per CYP2C19 status were excluded. The safety outcomes were major bleeding events, defined as symptomatic intracranial hemorrhage, intraocular hemorrhage causing vision loss, or other bleeding resulting in substantial hemodynamic compromise requiring treatment.^12^

### Study selection

Three assessors (F.C.C.K., J.v.d.B., and D.W.J.D.) independently screened the retrieved articles by title and abstract to identify studies that met the inclusion criteria. When discrepancies occurred with full-text screening of the articles, the discrepancy was discussed until consensus was reached. Conference abstracts were excluded. Studies with genotype-guided treatment allocation and trials that did not investigate the required treatment contrast were excluded after full text assessment. Since aspirin exerts its antiplatelet effect through cyclooxygenase (COX) inhibition instead of the P2Y12-receptor, it was therefore accepted as co-medication in the trials. If publications were based on the same clinical trial, only the most complete publication was included.

### Quality assessment and data extraction

Relevant data were extracted from each trial included in the meta-analysis. The following data were extracted: authors, year of publication, substudy, enrollment, participants, ancestry, number of participants in substudy, reported LoF carriers, *CYP2C19* variants, median follow-up period, outcomes, safety outcome, treatment, and blinding.

The assessors evaluated the risk of bias of each included trial by using the Cochrane Collaboration Risk of Bias assessment tool.^13^ The following domains were assessed: random sequence generation, allocation concealment, blinding of participants and personnel, blinding outcome assessment, incomplete outcome data, selective outcome reporting, and other sources of bias. Items were scored as “low risk of bias,” “some concerns,” or “high” risk of bias (Supplementary Material II).

### Statistical analysis

We estimated the effect of the investigated antiplatelet agent in each study, based on the reported numbers of outcomes (IS, major bleeding, and MACE) in the intervention and control groups. Interaction of *CYP2C19* was estimated by including an interaction factor between treatment and *CYP2C19* variant subgroup in a random effect regression analysis to assess for heterogeneity. The interaction analysis was performed on all trials, which were pooled to assess across-trial interaction only. However, this method may attribute differences between trials to patient characteristics without considering other variables. Therefore, a sensitivity analysis was performed based on the interaction effect of all trials separately to assess within-trial interaction alone to avoid ecological bias between studies.^14^ The total effect of *CYP2C19* LoF allele on treatment effect was then pooled by weighing the sample size of each trial and estimating a random effects model with the separately calculated interaction effects.^14^ The interaction term was considered statistically significant at p < 0.05. In addition, the size and direction of the interaction were evaluated through exponentiating the interaction coefficient to assess interaction effect of the CYP2C19 interaction on clopidogrel treatment effects. An exponentiated interaction term larger than 1 quantifies that the effect in both subgroups is not perfectly additive but multiplicative and differs between the two subgroups.^15^

All results are reported as odds ratios (OR) with 95% confidence intervals (CI) and are presented in forest plots. Heterogeneity was assessed with the I^2^ statistic to evaluate the variance between the studies per outcome for carriers and non-carriers separately. Analyses were stratified by the cardiovascular disease population and the TIA or minor stroke population.

Statistical analyses were performed with R Studio statistical software version 4.4.2. The following packages were used in the analysis: “*glm2*,” *“rms,” “readxl*,” *“lme4,” “meta*,” *“metafor,” and “forestplot.*”

## Results

### Study selection

**Figure 1.**
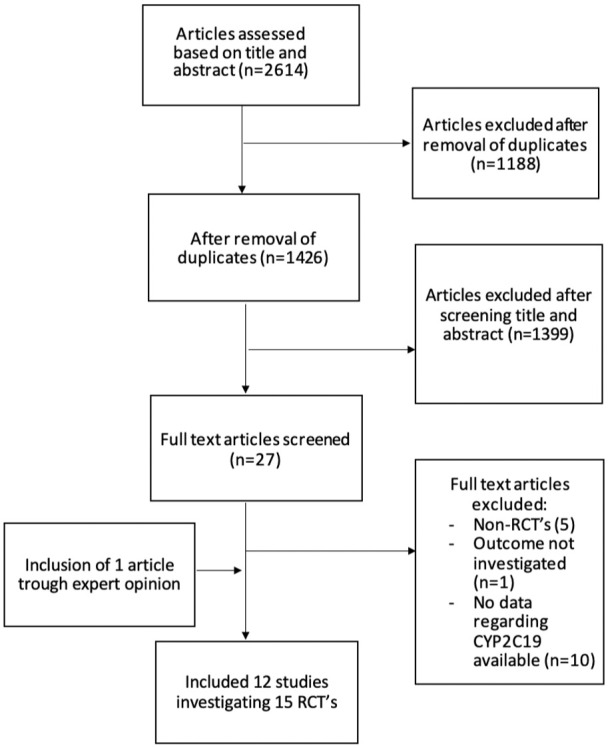
Flowchart of selection procedure.

The search provided 2614 articles for possible inclusion. After screening on title and abstract, 31 articles remained for full-text assessment. In the end, 12 eligible articles describing 15 RCTs identified and included in the meta-analysis (Figure 1 and Table 1).^5,16–26^

**Table 1.** Study characteristics of the included trials.

Study	Enrollment period	Year of publication	Patient population	Region	Participants with CYP2C19 genotyping (n)	LoF reported (n)	CYP2C19 variants	Follow-up time	Treatment given on background of aspirin	Loading + maintenance dose clopidogrel, ticagrelor, prasugrel (mg)	Blinding
Clopidogrel vs. placebo											
CHANCE^5^	Oct 2009 to Jul 2012	2016	Acute minor IS, TIA	Asian	2933	1726 (58.8%)	*2, *3, *17	90 days	Yes	300 + 75	Double
POINT^16^	May 2010 to Dec 2017	2020	Acute minor IS, TIA	White (70.8%), Afro/Black-Americans (23.2%), other (6.0%)	932	265 (28.4%)	*2, *3, *17	90 days	Yes	600 + 75	Double
CURE^17^	Dec 1998 to Sep 2000	2010	ACS without ST-segment elevation	European (85.9%), Latin American (14.1%)	5030	1325 (26.3%)	*2, *3, *17	1.91 years	Yes	300 + 75	Double
ACTIVE-A^17^	Jun 2003 to May 2006	2010	AF	European	1139	279 (24.5%)	*2, *3, *17	3.6 years	Yes	NI + 75	Double
CHARISMA^18^	Oct 2002 to Nov 2003	2012	CAD, Multiple atherothrombotic risk factors, PAD*, cerebrovascular disease	European	4537	1006 (22.2%)	*2, *3, *17	2.3 years	Yes	NI + 75	Double
Yi et al.^19^	Aug 2009 to Dec 2011	2018	IS	Asian	570	257 (45.1%)	*2, *3	5 years	Yes	NI + 75	NI*
Total no. of patients					15,141						
Clopidogrel vs. ticagrelor											
Dong et al.^20^	Aug 2013 to Mar 2014	2016	ACS	Asian	166	96 (58%)	*2, *3, *4, *8	1 month	No	600 + 75 vs. 180 + 2x 90	NI*
PLATO^21^	Oct 2006 to Jul 2008	2010	ACS	European	10,285	2772 (27%)	*2, *3, *4, *5, *6, *7, *8, *17	6–9 months	No	300 − 600 + 75 vs. 180 + 2x 90	Double
PRINCE^22^	Aug 2015 to Mar 2017	2019	Minor IS or high risk TIA	Asian	675	374 (58%)	*2,*3	3 months	Yes	300 + 75 vs. 180 + 2x 90	End-point blinded
Mohareb et al.^23^	April 2017 to Oct 2017	2020	ACS undergoing PCI	North African	963	148 (15%)	*2,*3	12 months	No	600 + 75 vs. 180 + 2x 90	Blinded treatment allocation
Total no. of patients					12,089						
Clopidogrel vs. prasugrel											
PRASFIT-ACS^24^	Dec 2010 to Jun 2012	2015	Patients scheduled for coronary stenting	Asian	773	485 (63%)	*2,*3	14 days	Yes	300 + 75 vs. 20 + 3.75	Double
TRITON-TIMI 38^25^	Nov 2004 to Jan 2007	2010	Moderate-to-high risk ACS	North American (32%), European (51%), Middle East, Africa, or Asia-Pacific (14%), South America (4%)	2943	802 (27%)	*2, *3, *4, *5, *6, *7, *8	6-15 months	Yes	300 + 75 vs. 60 + 10	Double
PRASTRO-I^26^	Sept 2011 to Jun 2015	2019	Non-cardioembolic IS	Asian (Japan)	3461	2298 (66%)	*2, *3	96–104 weeks	No	75 vs. 3.75**	Double
PRASTRO-II^26^	Sept 2012 to Oct 2014	2020	Non-cardioembolic IS with age ⩾75 years and/or weight ⩽50 kg	Asian (Japan)	586	400 (68%)	*2, *3	48 weeks	No	50 vs. 3.75 or 2.50**	Double
PRASTRO-III^26^	Oct 2018 to Apr 2020	2023	Non-cardioembolic IS with risk factors (hypertension, diabetes, chronic kidney disease, dyslipidemia, history of IS)	Asian (Japan)	196	125 (64%)	*2, *3	24–48 weeks	No	75 vs. 3.75**	Double
Total no. of patients					7959						

* NI = no information mentioned; ** No information whether loading doses were administered.

These RCTs included a total of 35,189 patients who were genotyped with at least one *CYP2C19* LoF variant allele (Table 1 and Supplementary Table II). A total of 15,141 patients were included in trials in which clopidogrel versus no clopidogrel was studied, 12,089 patients were included in studies that investigated clopidogrel versus ticagrelor treatment, and 7,959 patients were included in studies that investigated clopidogrel versus prasugrel treatment.

### Study characteristics

In seven of the 15 studies, the index event was a TIA or minor stroke,^5,16,19,22,26^ while the remaining eight studies included patients with cardiovascular disease.^17,18,20,21,23–25^ Eight studies were executed in Asian countries,^5,19^ six studies mainly recruited patients in Europe or North America,^17,18,27^ and one study was executed in North Africa.^23^ Six studies investigated clopidogrel versus no clopidogrel treatment in combination with a background treatment of aspirin,^5,17–19,27^ four studies investigated ticagrelor versus clopidogrel treatment,^20–23^ and five studies investigated prasugrel versus clopidogrel treatment.^24–26^ All studies assessed MACE, five studies reported ischemic stroke as outcome, and 12 studies reported major bleeding events (Supplementary Table II).

The median follow-up period varied from 14 days to 5 years (Table 1).

### Quality assessment

**Figure 2.**
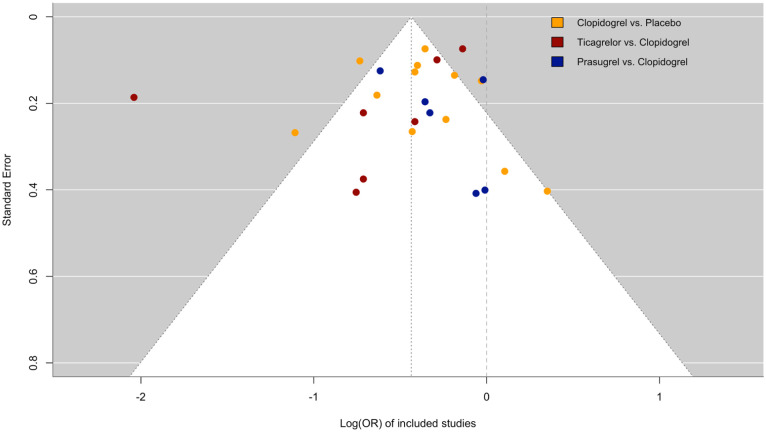
Funnel plot for publication bias in included studies.

Publication bias was low in most of the included studies (funnel plot, Figure 2), with the exception of one study.^23^ Risk of bias according to the Cochrane Risk of Bias assessment was low in most included trials for the following domains: random sequence generation, allocation concealment, blinding outcome assessment, incomplete outcome data, and other sources of bias (Supplementary Table III). In three studies, however, the risk of bias was high in one or more domains^20,26^.

### Effect of CYP2C19 on MACE

**Figure 3.**
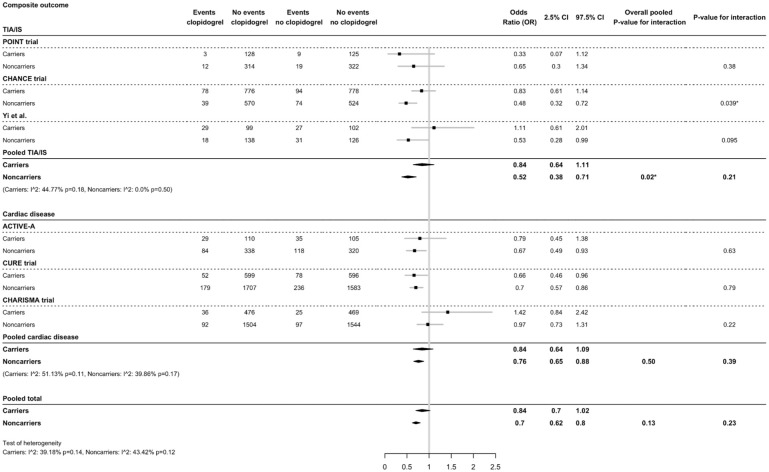
Effect of *CYP2C19* variant alleles and clopidogrel treatment on composite major cardiovascular events (MACE). Clopidogrel treatment is compared to placebo treatment on a background of aspirin.

The interaction effect of clopidogrel versus placebo for the total population on MACE was 1.21 times larger in non-carriers (OR = 0.70, 95% CI = 0.62-0.80) than in carriers (OR = 0.84, 95% CI = 0.70-1.02), but without significant interaction (p-interaction = 0.23) (Figure 3). Carriers showed small to moderate heterogeneity (I^2^= 39.18%, p = 0.14), while non-carriers showed moderate heterogeneity (I^2^= 43.42%, p = 0.12) (Figure 3). In TIA or minor stroke patients only, the interaction effect of clopidogrel on MACE was 1.36 times larger in non-carriers (OR = 0.52, 95% CI = 0.38-0.71) than in carriers (OR = 0.84, 95% CI = 0.64-1.11) (p-interaction = 0.21). The interaction effect of clopidogrel on MACE in the cardiovascular subgroup was larger in non-carriers (OR = 0.76, 95% CI = 0.65-0.88) than in carriers (OR = 0.84, 95% CI = 0.64-1.09) and was not significant (p-interaction = 0.39). Overall, carriers had a larger risk of MACE compared to non-carriers (Figure 3, Supplementary Figure I, and Supplementary Table IV).

**Figure 4.**
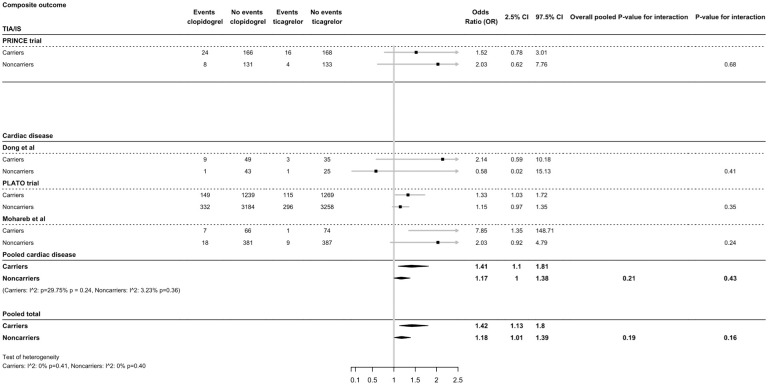
Effect of *CYP2C19* variant alleles and ticagrelor treatment on composite major cardiovascular events (MACE). Ticagrelor treatment is compared to clopidogrel treatment.

For patients treated with clopidogrel versus ticagrelor, the risk of MACE was 1.27 times higher in carriers (OR = 1.42, 95% CI = 1.13-1.8) compared to non-carriers (OR = 1.18, 95% CI = 1.01-1.39, p-interaction = 0.16) (Figure 4). For patients with cardiovascular disease only, the risk of MACE was higher in carriers (OR = 1.41, 95% CI = 1.1-1.81) compared to non-carriers (OR = 1.17, 95% CI = 1-1.38, p-interaction = 0.43). Heterogeneity among the included studies in the meta-analysis was small to moderate (Figure 4).

**Figure 5.**
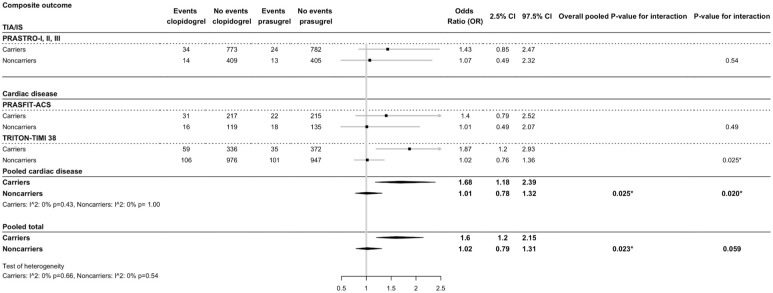
Effect of *CYP2C19* variant alleles and prasugrel treatment on composite major cardiovascular events (MACE). Prasugrel treatment is compared to clopidogrel treatment.

For patients treated with clopidogrel versus prasugrel, the risk of MACE was 1.56 times higher in carriers (OR = 1.6, 95% CI = 1.2-2.15) compared to non-carriers (OR = 1.02, 95% CI = 0.79-1.21, p-interaction = 0.059). For patients with cardiovascular disease only, carriers had a larger effect of prasugrel treatment compared to clopidogrel treatment (OR = 1.68, 95% CI = 1.18–2.39) versus non-carriers (OR = 1.01, 95% CI = 0.78-1.32, p-interaction = 0.02) (Figure 5).

**Figure 6.**
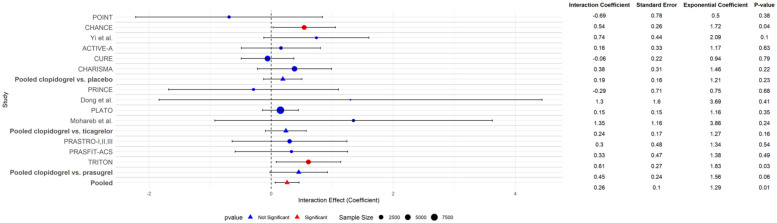
Overview of all CYP2C19 interactions for each treatment contrast (clopidogrel vs. placebo, clopidogrel vs. ticagrelor, and clopidogrel vs. prasugrel) and their pooled results on the outcome of MACE. Pooled interaction effects are calculated by weighing each interaction effect separately to assess within-trial interaction and weighed on their sample size to avoid ecological bias in heterogeneous studies.^14^ In the exponential coefficient column, the magnitude of the interaction effect of CYP2C19 on the outcome of MACE is described. Trials are grouped by treatment contrast (clopidogrel vs. placebo, clopidogrel vs. ticagrelor, clopidogrel vs. prasugrel), pooled per treatment contrast, and pooled for the total effect across all trials.

When all interaction effects are pooled, the total occurrence of MACE for variant carriers is 1.29 times higher compared to non-carriers and is statistically significant (p-interaction = 0.01) (Figure 6).

### Effect of CYP2C19 on ischemic stroke

The interaction effect of clopidogrel versus placebo on IS for patients with IS was 1.90 times larger in carriers (OR = 0.79, 95% CI = 0.58–1.08) than in non-carriers (OR = 0.51, 95% CI = 0.36-0.73) without a significant interaction effect (p-interaction = 0.64) (Supplementary Figure I).

No significant heterogeneity was measured among the included studies for carriers (p = 0.17) and non-carriers (p-interaction = 0.56).

### Effect of CYP2C19 on major bleeding

The interaction effect of clopidogrel versus placebo on major bleeding was smaller in non-carriers (OR = 1.27, 95% CI = 1.00-1.61) than in carriers (OR = 2.48, 95% CI = 1.57-3.83) without significant heterogeneity among the included studies (carriers: I^2^ = 16.62%, p = 0.31, non-carriers I^2^= 0%, p = 0.56, Supplementary Figure I).

In the cardiovascular disease population, the risk of major bleeding for clopidogrel treatment was smaller in non-carriers (OR = 1.28, 95% CI = 1.01-1.62) than in carriers (OR = 2.36, 95% CI = 1.49-3.83) (Supplementary Figure I). Because of the low number of bleeding events in the included trials, the risk of major bleeding in patients with TIA or minor stroke could not be determined. The interaction effect could not be reliably measured because of the low number of events.

For patients treated with ticagrelor versus clopidogrel, the risk of major bleeding was smaller in carriers (OR = 0.97, 95% CI = 0.76-1.23) compared to non-carriers (OR = 1.05, 95% CI = 0.89-1.23), with a non-significant interaction (Supplementary Figure II).

For patients treated with prasugrel versus clopidogrel, the risk of major bleeding was smaller in non-carriers (OR = 0.69, 95% CI = 0.49-0.96) compared to carriers (OR = 0.97, 95% CI =0.68-1.39, p = 0.28). For patients with cardiovascular disease only, the risk of major bleeding was not significantly different between carriers and non-carriers (Supplementary Figure III).

When all interaction effects are pooled, the total occurrence of major bleeding for variant carriers is higher compared to non-carriers but is not statistically significant (p-interaction = 0.99) (Supplementary Figure IV).

## Discussion

In this systematic review and meta-analysis, we evaluated how *CYP2C19* LoF allelic variants influence the efficacy of different antiplatelet agents on the occurrence of major vascular events or major bleeding in patients with a recent TIA, minor stroke, or cardiovascular disease. After combining data from 15 studies, the protective effect of clopidogrel on the occurrence of MACE is diminished due to the *CYP2C19* LoF variant. When the CYP2C19 interaction size and direction are pooled for all treatment contrasts, the interaction of CYP2C19 on clopidogrel is statistically significant. In addition, CYP2C19 LoF carrier status showed a statistically significant interaction on prasugrel versus clopidogrel treatment, as patients with an LoF carrier status seemed to benefit more from prasugrel treatment. This may partly be attributed to the diminished effect of clopidogrel treatment for patients with a *CYP2C19* LoF allele.

The association between clopidogrel effect and *CYP2C19* LoF variants has been investigated in other settings.^28–31^ Only one previous meta-analysis assessed the influence of *CYP2C19* LoF variants on TIA and minor stroke patients.^7^ In all of these meta-analyses, the beneficial effect of clopidogrel treatment on ischemic events in patients with an LoF variant was smaller than in patients without an LoF variant. However, these meta-analyses included RCTs, case-control studies, and cohort studies, which make them difficult to interpret. In our study, we only included RCTs, in order to minimize confounding and selection bias. In addition, most of the published meta-analyses did not assess interaction to assess the unbiased effect of a *CYP2C19* LoF variant on clopidogrel efficacy with random effects modeling.

Ticagrelor may have a larger inhibitory effect on platelets compared to prasugrel treatment.^32^ For patients with an ischemic stroke or TIA, various studies showed that ticagrelor treatment may lead to a higher risk of bleeding events compared to clopidogrel treatment. However, in large clinical trials, this higher risk could not be identified and ticagrelor seemed to possess a similar bleeding profile risk compared to aspirin treatment.^33–36^

In our study, non-carriers of the *CYP2C19* LoF allele showed a trend of a higher bleeding risk, but the interaction did not show statistical significance when interaction was investigated separately per trial (Supplementary Figure IV). Earlier studies showed lower bleeding rates in patients with a *CYP2C19* LoF variant.^7,37,38^ From a pathophysiological viewpoint, it is unlikely that patients with a *CYP2C19* LoF variant experience higher bleeding risks while on clopidogrel treatment. The trend in higher risk of bleeding for carriers of the *CYP2C19* LoF allele may be explained by the small number of events per treatment group, also indicated by the large CIs in Supplementary Figure IV, and needs to be interpreted with caution. It may also be possible that more patients of Asian descent were included in the carriers’ arm of the trials, since those patients more often possess a *CYP2C19* LoF allele. These patients may have other characteristics that may influence bleeding risk, such as a lower body weight or other CYP enzyme genetic profile differences. However, it is most likely that the direction of the relation is caused by chance, related to the low number of events per treatment group in the included trials.

Our study population consisted of participants with different manifestations of vascular disease and therefore different pathophysiological mechanisms leading to ischemic events. Clopidogrel, ticagrelor, and prasugrel have platelet aggregation lowering properties through inhibition of the P2Y12-receptor on platelets, which prevents thrombus formation in the blood vessels. CYP2C19 is also involved in prasugrel treatment but only has a small role in activating the prodrug of prasugrel.^39^ For ticagrelor, CYP2C19 metabolism is not involved. Compared to cardiovascular ischemia, a minor ischemic stroke or TIA is more often caused by a blockage of the blood vessel with a thrombo-embolus.^40^ Cardiovascular ischemia, on the contrary, is more often caused by reduced coronary artery perfusion due to local atherosclerosis.^41,42^ The difference in pathophysiology and the point of application of the active substances of clopidogrel may explain why no significant interaction effect was found in the cardiovascular disease population, even though a significant relationship between *CYP2C19* LoF variants and clopidogrel effectiveness has been observed in other studies for patients with cardiovascular disease.^43^

Part of the heterogeneity in the interaction effect may be explained by trial characteristics. In our meta-analysis, the length of the follow-up period varied from 14 days to 5 years. In addition, some trials investigated Asian populations, others investigated European populations, and some trials investigated mixed populations (Table 1). Since trials did not specify further what the ethnicity of the trial population was for poor, intermediate, and normal metabolizers separately, we unfortunately could not perform a subgroup analysis in our meta-analysis.

In addition to the available literature on the relationship of *CYP2C19* LoF variants and clopidogrel efficacy, multiple studies have been performed to investigate the implementation of genotype-guided therapy.^43–45^ These studies have mainly been performed in patients with a history of cardiovascular disease and show promising, although not conclusive, results regarding implementation of genotype-guided treatment.^43–48^ In the CHANCE-2 trial, minor stroke patients with a *CYP2C19* LoF mutation showed a better response on treatment with ticagrelor and aspirin compared to treatment with clopidogrel and aspirin.^45^

More studies on genotype-guided treatment for patients with cardiovascular disease as well as patients with a recent TIA or minor stroke and the implementation in real clinical practice are needed.

In the United States, clopidogrel is prescribed as a secondary prevention agent less frequently than in Europe. This may partly be due to the differences in the cost of clopidogrel in both regions. In addition, the costs of alternative antiplatelet agents vary greatly, mainly due to some antiplatelet agents, such as ticagrelor and prasugrel, still being subject to active patents. Ticagrelor and prasugrel are currently mainly prescribed as secondary prevention agents in patients with cardiovascular disease. Although the amount of research on the effectiveness and safety of these agents in patients with ischemic stroke is increasing, these agents are still more expensive than the generic variant of clopidogrel. Most of the current research on prasugrel has been performed in Asian populations.^26^ More studies regarding cost-effectiveness of prasugrel treatment are needed, especially in lower- and middle-income countries, since testing for CYP2C19 status may have higher associated costs than alternative treatment options.

A recent American Heart Association (AHA) statement addressed that testing for CYP2C19 status is recommended for patients with ACS.^49^ AHA or European Society of Cardiology (ESC) guidelines for ischemic stroke do not yet address CYP2C19 genotyping, nor does the (European Stroke Organization) (ESO) guideline on antithrombotic treatment for stroke prevention.^50^ Future studies regarding cost-effectiveness and effect in real-life settings of *CYP2C19* genotype-guided testing may provide insight into its prevention of recurrent ischemic events.

## Limitations

Our study has several limitations. All studies were performed as a post hoc analysis. Patients were genotyped for inclusion in a genetic substudy of the original trial. In most studies, the genotype had been determined in only a small proportion of patients from the original trial. For example, only one-fifth of the participants in the POINT and ACTIVE-A trials were genotyped for *CYP2C19* LoF variants.^16,17^ However, as the trials were all randomized, selection and confounding bias is unlikely.

Patients with one *CYP2C19* LoF allele and one functioning allele are considered intermediate metabolizers, whereas poor metabolizers have very low conversion of clopidogrel to its active metabolite by CYP2C19. When patients have suboptimal conversion of clopidogrel and therefore less effective platelet inhibition, these patients may benefit from a higher loading and maintenance dose of clopidogrel.^51,52^ However, no consensus exists on the effectiveness of a higher maintenance dose of clopidogrel for patients with a *CYP2C19* LoF variant allele. The absence of a differential effect of CYP2C19 in the POINT trial may have been caused by the higher clopidogrel loading dose of 600 mg that was employed.^53^ This higher loading dose could have prevented more events in the first days compared to the CHANCE trial, which could be one of the causes of the difference between the results of these trials.

In the present study, only carriers versus non-carriers of the *CYP2C19* allele were analyzed. We did not separately investigate poor and intermediate metabolizers of CYP2C19, since the number of events for each outcome within the poor metabolizer group was very small in all included studies. However, the consequences of these findings are comparable for intermediate and poor metabolizers, but the effect may be smaller in intermediate metabolizers.

In the CHANCE trial, approximately 25% of included patients had a TIA as their index event compared to approximately 40% in the POINT trial.^5^ Since, in most trials, the baseline National Institutes of Health Stroke Scale (NIHSS) was low, caution is needed when extrapolating these results to real clinical practice, where patients often experience more disabilities at presentation.

## Conclusion

Our meta-analysis shows that clopidogrel is less effective in patients with a recent MI, minor stroke, or TIA and a *CYP2C19* LoF genotype. The size and direction of the difference in effect warrant further research into the role of LoF genotypes and the cost-effectiveness of testing. Patients with a *CYP2C19* LoF genotype may benefit more from treatment with prasugrel or ticagrelor instead of clopidogrel, and therefore, it could be a viable alternative treatment option for both patients with cardiovascular disease and ischemic stroke or TIA.

## Supplemental Material

sj-docx-1-wso-10.1177_17474930251372371 – Supplemental material for Interaction of CYP2C19 with the effect of clopidogrel in secondary prevention of major ischemic events: Systematic review and meta-analysis Supplemental material, sj-docx-1-wso-10.1177_17474930251372371 for Interaction of CYP2C19 with the effect of clopidogrel in secondary prevention of major ischemic events: Systematic review and meta-analysis by Femke CC Kremers, Jochem van den Biggelaar, Hester F Lingsma, Ron HN van Schaik, Bob Roozenbeek and Diederik WJ Dippel in International Journal of Stroke
